# Supplementary material for: Systematic review on biomarker potential of vitreous microRNA in retinal disease
Source: Front Ophthalmol (Lausanne). 2025 Dec 15;5:1671266. doi: 10.3389/fopht.2025.1671266 (PMC12745263; doi:10.3389/fopht.2025.1671266)
Supplement: Supplementary file 1 [file DataSheet1.pdf]

Supplemental Materials

Article Title: Biomarker potential of vitreous microRNA in retinal disease: a meta-analysis

Journal: Discover Applied Sciences

Authors: Diana Joseph[1], Brian Grover[1], Michael Telias[1].

Affiliations: [1]University of Rochester Medical Center, Flaum Eye Institute, Rochester, NY, USA.

Corresponding Author: Michael Telias, Michael_Telias@urmc.rochester.edu

Supplementary Table 1. The 19 most commonly dysregulated (≥3 instances of dysregulation) miRNA species in proliferative diabetic retinopathy.

**miRNA**	**Study**	**Arm**	**Up/Downregulation**
miR-142	Kot 2022	miR-142-3p	Up
	Friedrich 2020	miR-142-3p	Up
	Kot 2022	miR-142-5p	Up
	Guo 2021	miR-142-5p	Up
miR-423	Guo 2021	miR-423-3p	Up
	Hirota 2014	miR-423-5p	Up
	Smit-McBride 2020	miR-423-5p	Up
	Guo 2021	miR-423-5p	Up
miR-9	Kot 2022	miR-9-3p	Down
	Liu 2022	miR-9-3p	Up
	Kot 2022	miR-9-5p	Down
	Guo 2021	miR-9-5p	Up

let-7a	Smit-McBride 2020	let-7a	Up
	Guo 2021	let-7a-3p	Up
	Guo 2021	let-7a-5p	Up
let-7g	Smit-McBride 2020	let-7g	Up
	Kot 2022	let-7g-5p	Up
	Guo 2021	let-7g-5p	Up
miR-1287	Smit-McBride 2020	miR-1287	Down
	Guo 2021	miR-1287-3p	Up
	Guo 2021	miR-1287-5p	Up
miR-15a	Hirota 2014	miR-15a	Up
	Kot 2022	miR-15a-5p	Up
	Guo 2021	miR-15a-5p	Up
miR-16	Smit-McBride 2020	miR-16	Up
	Kot 2022	miR-16-5p	Up
	Guo 2021	miR-16-5p	Up
miR-181a	Guo 2021	miR-181a-2-3p	Up
	Guo 2021	miR-181a-3p	Up
	Guo 2021	miR-181a-5p	Up
miR-185	Smit-McBride 2020	miR-185	Up
	Friedrich 2020	miR-185-5p	Up
	Guo 2021	miR-185-5p	Up
miR-30d	Guo 2021	miR-30d-3p	Up
	Guo 2021	miR-30d-5p	Up

	Smit-McBride 2020	miR-30d-star	Down
miR-320a	Hirota 2014	miR-320a	Up
	Smit-McBride 2020	miR-320a	Up
	Guo 2021	miR-320a-3p	Up
miR-320b	Hirota 2014	miR-320b	Up
	Smit-McBride 2020	miR-320b	Up
	Guo 2021	miR-320b	Up
miR-326	Friedrich 2020	miR-326	Up
	Guo 2021	miR-326	Up
	Friedrich 2020	miR-326-5p	Up
miR-425	Guo 2021	miR-425-3p	Up
	Guo 2021	miR-425-5p	Up
	Smit-McBride 2020	miR-425-star	Up
miR-486	Guo 2021	miR-486-3p	Up
	Smit-McBride 2020	miR-486-5p	Up
	Guo 2021	miR-486-5p	Up
miR-92a	Smit-McBride 2020	miR-92a	Up
	Kot 2022	miR-92a-3p	Up
	Guo 2021	miR-92a-3p	Up
miR-93	Hirota 2014	miR-93	Up
	Mammadzada 2019	miR-93	Up
	Guo 2021	miR-93-5p	Up
miR-223	Guo 2021	miR-223-3p	Up

	Guo 2021	miR-223-5p	Up
	Smit-McBride 2020	miR-223-star	Down

Supplementary Table 2. The 25 most commonly dysregulated (≥3 instances of dysregulation) miRNA species in proliferative vitreoretinal disease.

**miRNA**	**Study**	**Disease***	**Arm**	**Up/Downregulation**
miR-9	Kot 2022	PDR	miR-9-3p	Down
	Liu 2022	PDR	miR-9-3p	Up
	Kot 2022	PDR	miR-9-5p	Down
	Guo 2021	PdR	miR-9-5p	Up
	Toro 2020	RD with PVR	miR-9-5p	
miR-423	Guo 2021	PDR	miR-423-3p	Up
	Hirota 2014	PDR	miR-423-5p	Up
	Smit-McBride 2020	PDR	miR-423-5p	Up
	Guo 2021	PDR	miR-423-5p	Up
	Usui-Ouchi 2016	PVRD	miR-423-5p	Up
miR-139	Smit-McBride 2020	PDR	miR-139-3p	Up
	Guo 2021	PDR	miR-139-3p	Up
	Usui-Ouchi 2016	PVRD	miR-139-5p	Down
	Toro 2020	RD with PVR	miR-139-5p	Up
miR-142	Kot 2022	PDR	miR-142-3p	Up

	Friedrich 2020	PDR	miR-142-3p	Up
	Kot 2022	PDR	miR-142-5p	Up
	Guo 2021	PDR	miR-142-5p	Up
	Smit-McBride 2020	PDR	miR-16	Up
	Usui-Ouchi 2016	PVRD	miR-16	Up
	Kot 2022	PDR	miR-16-5p	Up
	Guo 2021	PDR	miR-16-5p	Up
miR-21	Usui-Ouchi 2016	PVRD	miR-21	Up
	Toro 2020	RD with PVR	miR-21-3p	Down
	Kot 2022	PDR	miR-21-5p	Up
	Guo 2021	PDR	miR-21-5p	Up
miR-223	Guo 2021	PDR	miR-223-3p	Up
	Guo 2021	PDR	miR-223-5p	Up
	Toro 2020	RD with PVR	miR-223-5p	Down
	Smit-McBride 2020	PDR	miR-223-star	Down
miR-486	Guo 2021	PDR	miR-486-3p	Up
	Toro 2020	RD with PVR	miR-486-3p	Up
	Smit-McBride 2020	PDR	miR-486-5p	Up
	Guo 2021	PDR	miR-486-5p	Up
miR-92a	Smit-McBride 2020	PDR	miR-92a	Up
	Usui-Ouchi 2016	PVRD	miR-92a	Up
	Kot 2022	PDR	miR-92a-3p	Up
	Guo 2021	PDR	miR-92a-3p	Up

miR-204	Usui-Ouchi 2016	PVRD	miR-204	Down
	Guo 2021	PDR	miR-204-3p	Up
	Kot 2022	PDR	miR-204-5p	Down
miR-27a	Mammadzada 2019	PDR	miR-27a	Up
	Guo 2021	PDR	miR-27a-3p	Up
	Toro 2020	RD with PVR	miR-27a-5p	Down
miR-361	Kot 2022	PDR	miR-361-3p	Down
	Guo 2021	PDR	miR-361-5p	Up
	Toro 2020	RD with PVR	miR-361-5p	Up
let-7a	Smit-McBride 2020	PDR	let-7a	Up
	Guo 2021	PDR	let-7a-3p	Up
	Guo 2021	PDR	let-7a-5p	Up
let-7g	Smit-McBride 2020	PDR	let-7g	Up
	Kot 2022	PDR	let-7g-5p	Up
	Guo 2021	PDR	let-7g-5p	Up
miR-1287	Smit-McBride 2020	PDR	miR-1287	Down
	Guo 2021	PDR	miR-1287-3p	Up
	Guo 2021	PDR	miR-1287-5p	Up
miR-15a	Hirota 2014	PDR	miR-15a	Up
	Kot 2022	PDR	miR-15a-5p	Up
	Guo 2021	PDR	miR-15a-5p	Up
miR-16	Smit-McBride 2020	PDR	miR-16	Up
	Kot 2022	PDR	miR-16-5p	Up

	Guo 2021	PDR	miR-16-5p	Up
miR-181a	Guo 2021	PDR	miR-181a-2-3p	Up
	Guo 2021	PDR	miR-181a-3p	Up
	Guo 2021	PDR	miR-181a-5p	Up
miR-185	Smit-McBride 2020	PDR	miR-185	Up
	Friedrich 2020	PDR	miR-185-5p	Up
	Guo 2021	PDR	miR-185-5p	Up
miR-30d	Guo 2021	PDR	miR-30d-3p	Up
	Guo 2021	PDR	miR-30d-5p	Up
	Smit-McBride 2020	PDR	miR-30d-star	Down
miR-320a	Hirota 2014	PDR	miR-320a	Up
	Smit-McBride 2020	PDR	miR-320a	Up
	Guo 2021	PDR	miR-320a-3p	Up
miR-320b	Hirota 2014	PDR	miR-320b	Up
	Smit-McBride 2020	PDR	miR-320b	Up
	Guo 2021	PDR	miR-320b	Up
miR-326	Friedrich 2020	PDR	miR-326	Up
	Guo 2021	PDR	miR-326	Up
	Friedrich 2020	PDR	miR-326-5p	Up
miR-425	Guo 2021	PDR	miR-425-3p	Up
	Guo 2021	PDR	miR-425-5p	Up
	Smit-McBride 2020	PDR	miR-425-star	Up

miR-93	Hirota 2014	PDR	miR-93	Up
	Mammadzada 2019	PDR	miR-93	Up
	Guo 2021	PDR	miR-93-5p	Up

*PDR = proliferative diabetic retinopathy, RD = retinal detachment, PVR = proliferative vitreoretinopathy, PVRD = proliferative vitreoretinal disease
